# Enfermedad hemolítica del feto y del recién nacido por aloanticuerpos contra el antígeno M

**DOI:** 10.7705/biomedica.5930

**Published:** 2021-12-15

**Authors:** Marco Páez, María Jiménez, Ana Corredor

**Affiliations:** 1 Departamento de Patología y Laboratorio Clínico, Fundación Santa Fe de Bogotá, Bogotá, D.C., Colombia Departamento de Patología y Laboratorio Clínico Fundación Santa Fe de Bogotá Bogotá D.C. Colombia; 2 Servicio transfusional, Fundación Santa de Bogotá, Bogotá, D.C., Colombia Servicio transfusional Fundación Santa de Bogotá Bogotá D.C. Colombia; 3 Inmunohematología, Fundación Santa Fe de Bogotá, Bogotá, D.C., Colombia Inmunohematología Fundación Santa Fe de Bogotá Bogotá D.C. Colombia

**Keywords:** eritroblastosis fetal, incompatibilidad de grupos sanguíneos, prueba de Coombs, ictericia neonatal, hiperbilirrubinemia neonatal, antígenos de grupos sanguíneos, enfermedad hemolítica del feto y del recién nacido, Erythroblastosis, fetal, blood group incompatibility, Coombs test, jaundice, neonatal, hyperbilirubinemia, neonatal, blood group antigens

## Abstract

Hay pocos reportes de enfermedad hemolítica del feto y del recién nacido causada por aloanticuerpos contra el sistema de antígenos MNS, especialmente, porque los anticuerpos que se generan contra estos antígenos son del tipo IgM, los cuales tienen reactividad a temperaturas inferiores a los 37 °C, y, por lo tanto, no son de importancia clínica. A pesar de ello, se han reportado casos con presencia de anticuerpos anti-M de tipo IgG causantes de la enfermedad hemolítica del recién nacido e, incluso, casos de muerte intrauterina por incompatibilidad materno-fetal en el sistema MNS. El proceso hemolítico se asemeja al causado por los anticuerpos anti-Kell, con anemia progresiva por supresión hematopoyética que induce la destrucción de precursores hematopoyéticos en la médula ósea y ausencia de reticulocitos en la periferia.

Se reporta el caso de una mujer con 38,5 semanas de gestación, que presentó discrepancia en la hemoclasificación directa y en la inversa. Como resultado, el recién nacido fue positivo en la prueba de Coombs directa sin que existiera incompatibilidad ABO con la madre. La correlación de estos resultados llevó a la detección de un anticuerpo anti-M en el suero materno. El diagnóstico definitivo fue posible gracias a la discrepancia en la hemoclasificación de la sangre materna. A pesar de que los anticuerpos anti-M usualmente no desempeñan un papel importante en la enfermedad hemolítica perinatal, este caso resalta la importancia de determinar la presencia de diferentes anticuerpos que pueden ser de vital interés a la hora de prevenir resultados graves asociados con dicha condición. Además, abre la puerta a nuevas recomendaciones relacionadas con la tamización y el tratamiento temprano de la hemólisis en los recién nacidos.

## Abstract (en)

There are few case reports of hemolytic disease in fetuses and newborns (HDFN) caused by alloantibodies against the MNS blood group system. The reason for this dearth is that antibodies toward these antigens are usually IgM, which not only cannot cross the placental circulation but also react at temperatures below 37°C. They are, therefore, of minimal clinical importance. Nevertheless, cases have been reported in which the presence of anti-M IgG antibodies caused severe HDFN and even intrauterine death in the presence of maternal-fetal MNS incompatibility indicating that they could have a high clinical impact. The hemolytic pattern observed in these cases is similar to that caused by anti-Kell antibodies. Progressive anemia is mediated and developed through hematopoietic suppression inducing the destruction of bone marrow precursor cells with the resulting absence of reticulocytes in peripheral blood.

This occurred in the case of a woman at 38.5 weeks of gestation who showed a discrepancy between direct and reverse blood type determination. A direct Coombs test was performed on the newborn’s blood, which was positive in the absence of maternal-fetal ABO incompatibility. Further tests were performed and anti-M antibodies were found in the maternal serum screening. Our final diagnosis was largely due to discrepancy issues in maternal blood. Although anti-M antibodies do not usually play a significant role in HDFN, this case stresses the importance of identifying the presence of antibodies that can be crucial in preventing HDFN and lead to new recommendations for the screening and prompt treatment of hemolysis in newborns.

El sistema sanguíneo MNS, a excepción del antígeno S, es considerado clínicamente significativo en medicina transfusional dado que genera anticuerpos IgM y entraña el riesgo potencial de causar una reacción hemolítica posterior a la transfusión. La incidencia del antígeno M es del 75 % en la población mundial, en tanto que su incidencia como aloanticuerpo (alo-Ac) en donantes es de 1 en 2.500 individuos ^1^^-^^3^. Los aloanticuerpos del sistema MNS que ocurren de forma natural fueron descritos por Wolff, *et al*., en 1933 ^4^. Estos anticuerpos son del tipo IgM, parecen tener mayor prevalencia en infantes que en adultos, son producto de la reacción cruzada tras la exposición poblacional a virus y bacterias, y no tienen significado clínico. La presencia de los IgM anti-M ha sido reportada con mayor frecuencia que la de los anticuerpos del tipo IgG como causantes de discrepancias en la hemoclasificación inversa al presentar reactividad a temperaturas menores de 37 °C; se resuelven al incubar la reacción a 37 °C ^5^.

Los anticuerpos anti-M se identifican en el 9 a 10 % de las mujeres gestantes cuando el rastreo de anticuerpos irregulares (RAI) resulta positivo. Usualmente son del tipo IgM, por lo que no tienen la capacidad de atravesar la barrera placentaria ^6^, pero existen casos reportados de anticuerpos anti-M causantes de la enfermedad hemolítica del feto y el recién nacido. Entre los anticuerpos contra antígenos no-Rh causantes de esta enfermedad hemolítica, la frecuencia de los anti-M es muy baja; estos se agrupan con anticuerpos en grupos sanguíneos como el Lewis o el Duffy, e incluso, aquellos de reacción inespecífica, con prevalencias hasta de menos del 5 % en casos de aloinmunización ^7^. En un reporte de Suresh, *et al*., en India, en que se hizo el cribado de 2.060 mujeres gestantes, se documentaron 22 casos de aloinmunización materna con RAI positivo, de los cuales solamente uno correspondía a la aloinmunización causada por anti-M, es decir, apenas el 4,5 % de las mujeres con aloinmunización. Los aloanticuerpos anti-M de significado clínico para la enfermedad hemolítica perinatal son reactivos únicamente a 37 °C y son anticuerpos de isotipo IgG o IgM de amplio espectro térmico, que pueden afectar al feto con diversos grados de gravedad.

Son contados los reportes del anti-M como causante de la enfermedad hemolítica perinatal. Se presenta con anemia de aparición tardía y de gravedad progresiva, y la prueba de Coombs directa es negativa o levemente positiva al nacimiento. Se han descrito casos de hiperbilirrubinemia de gravedad variable, muerte intrauterina en embarazos repetidos e, incluso, anemia neonatal grave. En la enfermedad hemolítica del feto y del recién nacido causada por el anti-M, hay destrucción de precursores eritropoyéticos, más frecuente que en los glóbulos rojos maduros debido a una mayor expresión de los antígenos MNS en la superficie de dichos precursores. Esta hemólisis cursa con hiperbilirrubinemia y reducción de reticulocitos en la circulación periférica, lo cual compromete la capacidad de reconstitución medular para reponer la población eritrocitaria ^6^^,^^8^.

## Descripción del caso

Se presenta el caso de una mujer de 30 años de edad en su primer embarazo producto de fertilización *in vitro*, con 38,5 semanas de gestación según la ecografía y sin antecedentes gineco-obstétricos. Ingresó para una cesárea programada por desproporción cefalopélvica (el peso fetal estimado en el último control fue de 3.800 g). La mujer presentaba diabetes gestacional desde la semana 20, la cual se le había tratado con dieta y ejercicio. No informó de transfusiones previas y la hemoclasificación del padre era O RhD positivo.

**Figura 1:**
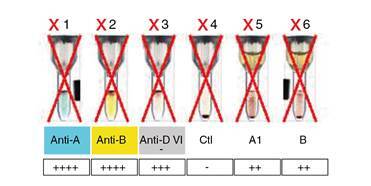
Hemoclasificación automatizada rechazada por el software debido a la discrepancia presente, lo que explica la X roja en cada pozo. En la prueba globular o directa (pozos 1, 2, 3 y 4), el grupo era claramente el AB. Sin embargo, en la prueba sérica o inversa (pozos 5 y 6), se identificaron anticuerpos anti-A y anti-B.

En el servicio transfusional se procesó una muestra materna para hemoclasificación, como parte del protocolo de obstetricia y ginecología (figura 1), con lo cual se evidenció la discrepancia entre la prueba globular y la sérica.

**Figura 2:**
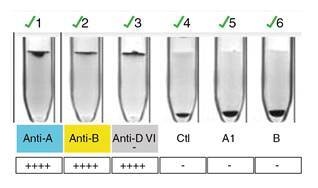
Hemoclasificación manual a 37 ^o^C . En la prueba globular o directa (pozos 1,2, 3 y 4), se confirmó la presencia de antígenos A y B sobre el eritrocito y, en la prueba sérica o inversa (pozos 5 y 6), se confirmó la ausencia de anticuerpos anti-A y anti-B vistos en los montajes a temperatura ambiente y a 4 ^o^C.

Tal como se observa en el cuadro 1 y la figura 2, la hemoclasificación directa y la inversa a 4 °C demostraron la presencia simultánea de antígenos A y B en los eritrocitos maternos con actividad anti-A y anti-B en el suero materno; al repetirse la prueba a 37 °C, la discrepancia ya no se presentaba al desaparecer la actividad de anticuerpos anti-A y anti-B.

**Cuadro 1:** La hemoclasificación manual a 37 o C (figura 2) resolvió la discrepancia y se concluyó que el grupo sanguíneo de la madre era AB RhD positivo

20 °C (temperatura ambiente)	4 °C	37 °C
Hemoclasificación directa:	Hemoclasificación directa:	Hemoclasificación directa:
Anti-A: 4+	Anti-A: 4+	Anti-A: 4+
Anti-B: 4+	Anti-B: 4+	Anti-B: 4+
Anti-D (VI-): 4+	Anti-D (VI-): 4+	Anti-D (VI-):4+
Control: negativo	Control: negativo	Control: negativo
Hemoclasificación inversa:	Hemoclasificación inversa:	Hemoclasificación inversa:
células A1: 2+	células A1: 2+	células A1: negativo
células B: 2+	células B: 2+	células B: negativo

**Figura 3:**
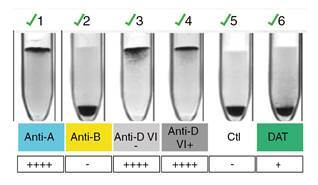
Hemoclasificación del recién nacido. Grupo sanguíneo A RhD positivo. En el pozo 6 se observa la prueba de Coombs con el resultado positivo de 1 +.

**Figura 4:**
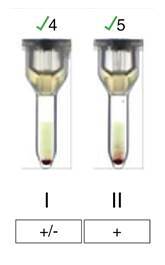
Rastreo de anticuerpos irregulares en la muestra materna. Las dos células fueron positivas, lo que hizo sospechar que la discrepancia se debía a un aloanticuerpo, que se confirmó posteriormente como un anti-M.

La recién nacida registró un peso de 3.485 g, APGAR de 8/9/10 y líquido amniótico claro, pues no hubo complicaciones durante la cesárea. El nacimiento fue a término, con peso adecuado y adaptación neonatal espontánea. La hemoclasificación de sangre de cordón umbilical fue: grupo sanguíneo A RhD positivo con prueba de Coombs directa positiva 1 + (figura 3). Al correlacionar este último resultado con la discrepancia inicial de la hemoclasificación materna, y en ausencia de incompatibilidad ABO entre la madre y la recién nacida, se decidió hacer un rastreo de anticuerpos irregulares (RAI) en la muestra materna, el cual resultó positivo (células I: positivo leve; células II: 1+) (figura 4). La muestra materna fue remitida a la unidad transfusional de referencia y allí se identificó un aloanticuerpo anti-M.

**Figura 5:**
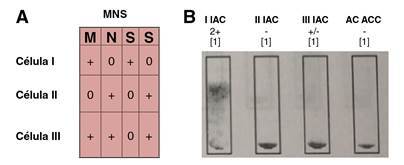
A. Panel de células de rastreo: fenotipo del grupo MNS. Con respecto al antígeno M, la tabla demuestra su expresión en las células I y III. B. El rastreo de anticuerpos irregulares y de autocontrol en la muestra materna dio resultados positivos para las células I y III. La reactividad con estas dos células concordó con la presencia de un anticuerpo de especificidad anti-M en el suero materno.

Posteriormente, en la muestra de la recién nacida, se analizó el fenotipo para el antígeno M del grupo sanguíneo MNS, con el fin de confirmar o descartar que la sensibilización observada en la Coombs directa positiva que se le practicó se debiera a la presencia del anticuerpo anti-M identificado en la muestra materna. Además, se analizó el fenotipo de la madre para confirmar que correspondiera a un antígeno M del sistema MNS negativo (figuras 5 y 6). El resultado en la unidad transfusional de referencia confirmó las dos premisas.

**Figura 6:**
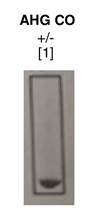
Fenotipo para el antígeno M del sistema MNS. Se confirmó la presencia del antígeno M sobre la superficie del glóbulo rojo de la recién nacida.

Se informó este hallazgo a la unidad de neonatos para que hicieran el seguimiento por riesgo de hemólisis. Dada la evolución satisfactoria de la recién nacida se tenía programada su salida, pero con la notificación de los resultados del laboratorio, se decidió dejarla en observación.

En los exámenes de laboratorio practicados se encontraron los siguientes resultados: hemoglobina, 16,7 g/dl; hematocrito, 48,4 %; leucocitos, 21,3 x 10^3^ /μΙ; neutrófilos, 68,5 %; linfocitos, 19,3 %; plaquetas, 349,000 por μΙ; glucosa, 56 mg/dl; bilirrubina total, 3,5 mg/dl, directa, 0,25 mg/dl, e indirecta, 3,25 mg/ dl, con lo cual se determinó que la intervención era innecesaria. A las 21 horas del nacimiento, las glucometrías eran normales, la succión adecuada y el tono muscular bueno. No presentaba dificultad respiratoria y la bilirrubina (*bilicheck*) estaba entre 5,3 y 5,7 mg/dl. Con estos resultados no se requería intervención y la bebé evolucionó satisfactoriamente, por lo cual se le dio salida.

Se empleó la columna de micropartículas (BioRad®) para determinar su movilidad y la lectura de los pozos se hizo en un sistema automatizado IH-500 de Biocientífica.

## Consideraciones éticas

El reporte del caso fue revisado y aprobado por el Comité Corporativo de Ética en Investigación de la Fundación Santa Fe de Bogotá (#CCEI-10817-2019).

## Discusión

Los antígenos del sistema MNS se expresan en la membrana eritrocitaria en las estructuras denominadas glucoforina A (GPA), glucoforina B (GPB) y las glucoforinas híbridas producto de recombinaciones genéticas de los genes *GPA* y *GPB*; estas últimas, las híbridas, son de mayor prevalencia en asiáticos y casi inexistentes en blancos. Las glucoforinas son glucoproteínas de paso único por la membrana, compuestas principalmente de ácido siálico y con gran número de puntos de glucosilación; este tipo de estructuras explican la naturaleza electronegativa de la superficie del eritrocito. Las glucoforinas interactúan con otras estructuras como la banda 3, las proteínas del sistema Rh, las acuaporinas, los transportadores de glucosa de tipo I y la espectrina, formando complejos que regulan el intercambio gaseoso y la estabilidad osmótica de la membrana eritrocitaria. La expresión de los genes *GPA* y *GPB* ocurre tempranamente en la hematopoyesis y representa uno de los primeros marcadores de linaje del compromiso eritroide ^2^.

Los antígenos expresados en glucoforinas híbridas se caracterizan por generar aloinmunización mediada por IgG, a diferencia de los fenotipos de mayor prevalencia en raza blanca (M y N), los cuales, tras las transfusiones o la gestación, generan de forma típica aloinmunización de naturaleza IgM. A pesar de ello, se han identificado anticuerpos anti-M y anti-N como causantes de reacciones hemolíticas después de la transfusión ^5^^,^^9^^,^^10^ y anemia del recién nacido, esta última, con graves consecuencias ^6^^,^^8^^,^^11^^,^^12^.

La prevalencia de este aloanticuerpo parece ser baja; se resalta que no todos los casos concluyen en enfermedad hemolítica del feto y del recién nacido, dada la naturaleza de tipo IgM del anticuerpo formado, por lo que es incapaz de atravesar la barrera feto-placentaria. Sin embargo, la preocupación aparece en aquellos casos de transfusiones o gestaciones que generan aloinmunización con el anti-M de tipo IgG. Los casos de enfermedad hemolítica perinatal causados por el anti-M, se caracterizan por la gran variabilidad de sus manifestaciones clínicas, por ejemplo, mujeres con antecedentes de abortos repetidos en gestaciones superiores a las 20 semanas ^6^^,^^12^, y neonatos con anemia progresiva e hiperbilirrubinemia ^6^, incluso en aquellos sin afectación evidente, prueba de Coombs directa levemente positiva y ausencia de anemia ^1^.

El caso que aquí se presenta fue detectado tras el estudio de una discrepancia en la hemoclasificación de la sangre materna, la cual se resolvió con la incubación a 37 °C, dando cuenta de un anticuerpo IgM de naturaleza fría que causaba aglutinación con las células A1 y B de la hemoclasificación inversa, sin tratarse de una reacción mediada por anticuerpos frente a los antígenos del sistema ABO. La prueba de Coombs directa positiva en el recién nacido obligó a ampliar el estudio para incluir este anticuerpo causante de la discrepancia, dada la ausencia de incompatibilidad ABO o Rh entre la madre y su hijo (madre AB RhD positivo, hijo A RhD positivo), lo que podría causar este resultado.

El rastreo y posterior identificación de anticuerpos irregulares en el suero materno, permitieron identificar la presencia del anti-M, el cual se reconoció como causante de la sensibilización *in vivo* de los eritrocitos de la recién nacida, cuyo fenotipo era positivo para el antígeno M. Además, se estableció que el anti-M descrito incluía ambas subclases de inmunoglobulinas, la IgM, causante principal de la discrepancia en la hemoclasificación materna dado el rango térmico en que se encontraba, y la IgG, capaz de atravesar la barrera feto-placentaria y causante de hemólisis en el recién nacido.

Dilucidado el caso, el servicio de neonatología fue informado de la posibilidad de que se presentara hemólisis y anemia del recién nacido, por lo que se hizo un seguimiento clínico y paraclínico, el cual evidenció únicamente un aumento en los valores de bilirrubinas que no ameritaba intervención.

## Conclusiones y recomendaciones

La enfermedad hemolítica del feto y del recién nacido tiene gran incidencia en casos de incompatibilidades antigénicas del grupo Rh entre madre y feto, con consecuencias graves en la mayoría de los casos. También, se ha descrito con otros grupos sanguíneos como el Kell, y menos frecuentemente, con el antígeno M del sistema MNS. En este último caso, las manifestaciones clínicas son muy variadas, con consecuencias de resolución espontánea la mayoría de las veces, sin que medie mucha intervención médica más allá del seguimiento paraclínico. Sin embargo, algunos de los casos descritos han sido más graves, lo que amplía el espectro del compromiso neonatal.

El presente caso evolucionó de forma benigna y fue diagnosticado a raíz de las discrepancias en la hemoclasificación de la madre, así como de la prueba de Coombs positiva en la recién nacida, a pesar de no tener una incompatibilidad evidente de grupo o de Rh. Esta circunstancia abre las puertas para la evaluación de nuevas recomendaciones con respecto a la importancia del estudio de los anticuerpos irregulares en el desarrollo de la enfermedad hemolítica del feto y del recién nacido, la cual puede representar un riesgo alto para niños recién nacidos a nivel mundial.

Este caso también evidencia que, cuando existe una discrepancia de hemoclasificación entre la prueba directa y la indirecta, la causa puede ser una incompatibilidad que debe ser estudiada para evitar posibles complicaciones. Otra prueba muy útil, que no se utilizó en este caso, es la identificación de anticuerpos en el eluido de la muestra del recién nacido, para constatar la identificación del anticuerpo sobre la superficie del eritrocito.
